# Leveraging the Domain of Work to Improve Migrant Health

**DOI:** 10.3390/ijerph14101248

**Published:** 2017-10-19

**Authors:** Michael A. Flynn, Kolitha Wickramage

**Affiliations:** 1 Occupational Health Equity Program Coordinator, National Institute for Occupational Safety and Health, Patriots Plaza 1, 395 E Street, SW, Suite 9200, Washington, DC 20201, USA; 2 Global Migration Health Research and Epidemiology Coordinator, International Organization for Migration (IOM), The United Nations Migration Agency, CH-1211 Geneva, Switzerland; kwickramage@iom.int

**Keywords:** migrant health, occupational health, social determinants of health, sustainable development, corporate sustainability

## Abstract

Work is a principal driver of current international migration, a primary social determinant of health, and a fundamental point of articulation between migrants and their host society. Efforts by international organizations to promote migrant health have traditionally focused on infectious diseases and access to healthcare, while international labor organizations have largely focused on issues of occupational health. The underutilization of the domain of work in addressing the health of migrants is truly a missed opportunity for influencing worker well-being and reducing societal economic burden. Understanding of the relationships among migration, work, and health would facilitate further integration of migrant health concerns into the policy agenda of governments and international agencies that work at the nexus of labor, health and development. The domain of work offers an opportunity to capitalize on the existing health and development infrastructure and leverage technical resources, programs and research to promote migrant health. It also provides the opportunity to advance migrant health through new and innovative approaches and partnerships.

## 1. Introduction

Work is a principle driver of current international migration [1]. Of the 232 million international migrants, an estimated 150.3 million are migrant workers, actively participating in the labor force of destination countries [1]. Surveys of irregular migrants—those entering, residing, and/or working in a country without proper authorization—have consistently shown employment as the major factor driving irregular routes of migration [2]. Even when migration is driven by safety concerns and political unrest, for example the recent wave of migration from North Africa to Europe, finding gainful employment in the host country remains a central concern for international migrants. Global estimates of migrant workers in 2016 reveal the vast majority to be employed within the service sector (71.1%), including 7.7% who work as domestic workers [1]. The services sector is followed by industries such as manufacturing/construction (17.8%) and agriculture (11.1%) [1].

Migration is a hallmark of the global economy, and migrant flows to both traditional and new destinations have continually increased over the past 30 years [1]. The United Nations (UN) identifies work—and more recently migration—as enablers of global development [3]. Indeed, for many low-income countries (e.g., Nepal, Liberia, Haiti, Tajikistan), migrant worker remittances form the largest source of foreign currency income, totaling US$429 billion in 2016—more than double the official development assistance provided by foreign states and international development organizations [4]. However, despite these economic contributions, the impact migration has on the health and well-being of international migrants and sending/receiving communities remains poorly understood and underrepresented in the research literature, especially within the area of low-skilled migrant workers in the Global South [5]. Under the Social Determinants of Health paradigm, the domain of work offers an opportunity to capitalize on the existing health and development infrastructure to better understand the relationship between migration, work, and health and advance migrant health through new and innovative approaches and partnerships.

### 1.1. Work and Migration as Social Determinants of Health

Social determinants of health (SDH) are defined as the conditions in which people are born, grow, live, work, and age [6]. Work is a primary way individuals meet the three fundamental needs that underpin action on health inequalities: the material requisites for a decent life; control over factors that influence their lives; and participation in society [7,8]. Migration can also have a significant impact on health. Most migrant workers occupy a vulnerable social position that can result in exploitative labor arrangements, limited access to resources, and declining health outcomes [9]. Migration also exposes labor migrants and their families to risk factors such as family separation, acculturative stress and discrimination that can negatively impact their physical, emotional, and social health [10]. Academics and international public health institutions have recently begun to acknowledge migration as a social determinant of health [10,11,12,13]. However, more research is needed to better understand the positive and negative impacts that work, migration and their interaction can have on the health of individuals in different settings and situations across the globe.

The World Health Organization’s (WHO) Commission on Social Determinants of Health (CSDH) increasingly integrates the SDH paradigm into international development frameworks, policy agendas and programs such as the UN initiatives around decent (safe, stable, and well-paid) work [11], sustainable development [3], and corporate sustainability [14]. The SDH paradigm provides a conceptual link between health and sustainable economic development. A greater recognition of the relationships among migration, work, and health [15] by public health and development professionals, could lead to a further integration of migrant health in the policy agendas of international labor, health and development organizations, and facilitate the inclusion of the domain of work into international efforts to document and improve the physical, mental, and social well-being of migrants.

### 1.2. Migration, Work, and Health

Despite differences in destinations and industries, the overwhelming majority of migrants are employed in what have come to be known as “3D”-dirty, demanding, and dangerous-jobs [16]. Whether it is increased rates of workplace injuries, such as the elevated incidence of fatal and non-fatal injuries among Latino migrant construction workers in the United States [17], exposure to physical and emotional abuse by employers as in the case of Sri Lankan women working as domestic maids in the Middle East [18,19], or exposure to hazardous materials such as silica dust among migrant gold miners in South Africa [20,21,22], the workplace is often a source of health risks for migrant workers.

The influence someone’s job or career has on their health goes beyond the physical, emotional and social risks that they face at work [8]. The strong link between work and migration [1] suggests that migration can be understood as part of a career path which exerts a significant influence over aspects of life outside the workplace that contribute or detract from a migrant worker’s health and that of their family [10,12,15]. For example, the crowded living conditions for migrant workers in the South African mining industry and the long periods of separation from their families are suggested to have contributed to epidemics of tuberculosis and HIV infection in Southern Africa [21,22]. Likewise, work can influences lifestyle changes, such as diet [23], which may contribute to the positive association between time in the United States and elevated rates of chronic diseases such as diabetes [24] among Latino migrants. It has been suggested that improving migrant health is largely dependent on upgrading working conditions [25]. 

## 2. Migration, Work, and Health in International Policy and Legal Frameworks

### 2.1. International Migration and Health Organizations

Efforts by international organizations to promote migrant health have traditionally focused on infectious diseases and access to healthcare [26,27,28,29]. The only health-related mention of migration in the UN 2000 Millennium Development Goals (MDG) Declaration was identifying its role in contributing to the global malaria problem [26]. The approach of international organizations and governments to migrant health is evolving. The WHO 2008 World Health Assembly Resolution (WHA 61.17) on the “health of migrants” acknowledged the inherent connection between migration and health outcomes, calling upon governments to address health concerns of migrants and mobile populations. The WHA resolution did not, however, specify the importance of leveraging the domain of work in addressing the health of migrants. 

An operational action framework for implementing the WHA resolution was formulated in May 2010 at the 1st Global Consultation (GC) in Migration Health led by the International Organization for Migration (IOM), WHO and the Government of Spain, which hosted the event [30]. The action framework articulated the need for countries to develop migrant-sensitive and mobility-competent health systems through inter-sectoral action and enabling domestic policy and legal frameworks on migrant health. While no specific recommendations were made on workplace settings as a crucial conduit/intervention space to address migrant health vulnerabilities, the framework did promote the need to engage a wide variety of stakeholders, including employers, in developing action plans on migrant health and determining what actions they should take among their own constituencies. Despite the robust action framework, the 2nd GC held in Sri Lanka in February 2017, organized by IOM, WHO, and the Government of Sri Lanka revealed major gaps in advancing the migration health agenda at the national, regional, and global levels [31]. The role and participation of governments, private sector industries, employer groups and trade unions, for instance, has been poorly mapped at national, sub-regional, regional and global levels [30]. 

### 2.2. International Development and Labor Organizations

International development and labor organizations largely limit their health-related efforts to issues of occupational safety and health. For example, the Global Plan of Action on Workers’ Health, Resolution WHA 60.26 identifies the need to pay particular attention to the safety of working conditions of migrant workers and other vulnerable working populations [32]. In addition, the UN Global Compact includes occupational health as a central element of corporate sustainability [14]. 

International efforts to improve migrant workers’ health have recently begun to expand beyond just focusing on workplace safety and health. For example, migrant workers were frequently mentioned in the Report on Employment Conditions and Health Inequalities to the WHO CSDH [8] and promoting fair employment and decent work is one of the 17 UN 2030 Sustainable Development Goals adopted in 2015 [3]. While this report did not delve into the relationships among migration, work, and health, it provides a more comprehensive conceptual model that expands the current paradigm beyond unsafe working conditions to include work as a central contributor to overall health and well-being. This broader understanding facilitates including the domain of work in international global efforts to document and improve migrant health. This could result in the recognition of the health related aspects of efforts to improve working conditions and secure better pay. It could also lead to innovative approaches to improving the health of migrant workers, both on and off the job, by using the workplace for prevention activities and other public health interventions. The possibility of a more holistic approach requires us to examine current practices and explore where they might overlap.

## 3. Building on Traditional Approaches

The field of occupational health has traditionally focused on eliminating the physical, chemical, biologic, and psycho-social hazards found at the workplace [7,8,33,34,35], while the field of migrant health has traditionally focused on infectious disease and access to health care [26,27,28,29]. Efforts to improve workplace safety and access to healthcare for migrants are essential and should continue. However, there is growing recognition that one’s job or career exerts a significant influence over other aspects of life that contribute or detract from workers’ health and that of their families [6,8]. This is part of a larger trend towards a more holistic and nuanced perspective on work and its impact on population health [6,33,35]. These new approaches are blurring the traditional boundaries between occupational and community health. Two additional areas of focus for occupational health have emerged: the relationships among job-related factors (e.g., wages, paid leave, job security) and health, and the utilization of the workplace as a venue for broader prevention and population health interventions [33,34]. 

The Total Worker Health^®^ program of the US National Institute for Occupational Safety and Health (NIOSH) is an operationalization of this expanded approach [35]. NIOSH defines Total Worker Health^®^ as policies, programs, and practices that integrate protection from work-related safety and health hazards with promotion of injury and illness prevention efforts to advance worker well-being. While efforts in these areas have largely centered on white-collar workers in high-income countries, there are examples of this approach being used to address non-communicable diseases among blue-collar workers in middle- and low-income countries [36]. These studies suggest that the overarching schema could be adapted for use with migrant workers in diverse geographic settings. A greater integration of migration, work and health in the policy frameworks of international organizations located at the nexus of health and development could help facilitate this adaptation. It is critical that such frameworks be adapted and applied to the migration dynamics of the respective national/regional contexts via inter-sectoral engagement from relevant stakeholders (e.g., labour, health, private sector, trade unions and civil society groups).

### 3.1. Improving Migrant Health Through Decent Jobs

Migration can provide access to greater financial resources but can also expose labor migrants and their families to risk factors such as economic insecurity, exploitative labor arrangements, and sub-standard housing that can negatively impact their physical, emotional, and social health [15]. For example, greater length of stay in the United States contributes to elevated rates of chronic illness [24] and mental illness among Latino migrants [37]. These declines are linked to lifestyle changes, such as unfavorable dietary habits, which respond to the demands of low-wage, contingent employment in the United States [25]. Access to stable, well paid, and safe work could help mitigate some of the lifestyle factors contributing to these declines and, ultimately, improve the health of migrant workers and promote sustainable economic growth [3,6,33]. Seen through this lens, the ILO efforts to include migrant workers in the Decent Work Agenda [11] can be understood as a migrant health initiative as it contributes to the overall health and well-being of migrant workers by attempting ensure an adequate standard of living through better work. 

Promoting decent work for migrants has the potential added benefit of addressing tensions between native and foreign-born workers. Many native-born workers also face growing economic insecurity and more precarious forms of employment. The ILO has long recognized that “lack of labor protection for migrant workers undermines protection generally for all workers” [11]. Efforts to improve working conditions for migrants would also likely involve and benefit native workers as well. This approach could help to break down barriers between these groups of workers, improve social integration, and promote a sense of a common cause among workers [38]. It also provides a model for further incorporation of migrant health into existing efforts by international bodies such as the ILO promotion of decent work [11], the UN agenda for sustainable development [3], and the UN Global Compact on corporate sustainability [14].

### 3.2. Improving Migrant Health Through Workplace Health Programs

Migrant workers often experience social marginalization and have limited access to the public health infrastructure of the host country [9]. Local health care and public health institutions often have not developed the institutional capacity to account for the specific needs and circumstances of migrant workers [39]. Irregular migration status can also result in legal and social exclusion from medical services [40,41]. As a result, migrant workers regularly face significant barriers to accessing health services and are frequently “invisible” to or considered “hard to reach” by public health institutions. Using the workplace as a forum to address broader public health concerns with migrant workers could help address some of these barriers. For example, while many migrants undergo mandatory medical examination to screen for diseases such as tuberculosis prior to or upon arrival to workplace settings, irregular migrants often do not [42]. Development of meaningful partnerships by public health organizations with employers, labor placement agencies, and health systems could help to ensure screening, continuity of care, and referral of positive cases for all labor migrants. It is important that migrant health initiatives adopt a holistic approach and not exclusively focus on infectious disease. Similar worksite programs could be designed for chronic diseases, such as diabetes or mental health concerns, such as social isolation and depression. 

Focusing on the workplace as a venue for comprehensive illness and injury prevention activities could facilitate the involvement and support of the private sector as labor migration fills a central need of employers for a reliable and healthy workforce. This focus would also help employers ensure the health of all of their employees by reducing the potential for disease transmission through identification and treatment of infected workers. A study on the feasibility of a workplace tuberculosis intervention for migrant workers suggests that obstacles to implementing migrant health interventions at the workplace could include fear of dismissal of employees with positive test results, potential productivity loss, employer liability, and stigmatization of migrants and the businesses where they work as a risk to public health [43]. To be successful, steps such as ensuring confidentiality of employee health records would have to be taken to address these barriers. Despite these obstacles, the workplace offers a central location that migrant workers return to on a daily basis, making it a recurring opportunity for public health interventions with “hard to reach” populations. 

## 4. Conclusions

The social determinants of health paradigm allows for a greater recognition of the relationships among migration, work, and health, and facilitates the integration of migrant health concerns into the policy agendas of governments and international agencies that work at the nexus of health, development and sustainability. Inclusion of migrant health into efforts such as the ILO decent work agenda [11], the UN agenda for sustainable development [3], and the UN Global Compact on corporate sustainability [14] could help leverage technical resources, programs, and research to forge partnerships to better understand and improve the health status of the world’s migrant workers. The domain of work also offers an opportunity to advance migrant health through partnerships with a coalition of government, trade and industry groups, employers, trade unions, and health actors. It is time for all of us within the global health, labor migration, and development sectors to collectively address the health of migrant workers.
